# Two different forms of inherited human TCRα chain deficiency

**DOI:** 10.70962/jhi.20250014

**Published:** 2025-06-04

**Authors:** Marie Materna, Simin Seyedpour, Tom Le Voyer, Nima Parvaneh, Niloufar Yazdanpanah, Amir Ali Hamidieh, Mehrzad Mehdizadeh, Hassan Rokni-Zadeh, Majid Changi-Ashtiani, Krishnajina Amarajeeva, Mana Momenilandi, Jean-Laurent Casanova, Mohammad Shahrooei, Jacinta Bustamante, Nima Rezaei, Vivien Béziat

**Affiliations:** 1 Laboratory of Human Genetics of Infectious Diseases, INSERM U1163, Necker Hospital for Sick Children, Paris, France; 2 Imagine Institute, Paris Cité University, Paris, France; 3 Research Center for Immunodeficiencies, Children’s Medical Center, Tehran University of Medical Sciences, Tehran, Iran; 4 Clinical Immunology Department, AP-HP, Saint-Louis Hospital, Paris, France; 5 Division of Allergy and Clinical Immunology, Department of Pediatrics, Children’s Medical Center, Tehran University of Medical Sciences, Tehran, Iran; 6 Network of Immunity in Infection, Malignancy and Autoimmunity (NIIMA), Universal Scientific Education and Research Network (USERN), Tehran, Iran; 7 Pediatric Cell and Gene Therapy Research Center, Gene, Cell & Tissue Research Institute, Tehran University of Medical Sciences, Tehran, Iran; 8 Pediatrics Center of Excellence, Children’s Medical Center, Tehran University of Medical Sciences, Tehran, Iran; 9 Department of Medical Biotechnology, School of Medicine, Zanjan University of Medical Sciences (ZUMS), Zanjan, Iran; 10 Specialized Immunology Laboratory of Dr. Shahrooei, Tehran, Iran; 11 St. Giles Laboratory of Human Genetics of Infectious Diseases, Rockefeller Branch, The Rockefeller University, New York, NY, USA; 12 Department of Pediatrics, Necker Hospital for Sick Children, AP-HP, Paris, France; 13 Howard Hughes Medical Institute, New York, NY, USA; 14 Department of Microbiology and Immunology, Clinical and Diagnostic Immunology, KU Leuven, Leuven, Belgium; 15 Center for the Study of Primary Immunodeficiencies, AP-HP, Necker Hospital for Sick Children, Paris, France

## Abstract

Genetic defects that result in the absence of all T cells, including both αβ and γδ T cells, are classified as severe combined immunodeficiency (SCID), a life-threatening condition requiring immediate hematopoietic stem cell transplantation (HSCT) in affected newborns. Previously, patients with a homozygous c.*+1G>A splice variant in the T cell receptor (TCR) α constant chain (*TRAC*) were found to lack only αβ T cells and demonstrated longer survival compared with SCID patients lacking both αβ and γδ T cells. This observation suggested that γδ T cells might partially compensate for the absence of αβ T cells. Here, we describe two children with biallelic premature stop codons in TRAC. These mutations result in a complete loss of TCRαβ expression on the cell surface and an absence of αβ T cells, leading to severe immunodeficiency and early death. Additionally, we demonstrate that the previously reported c.*+1G>A TRAC variant retains partial activity in vitro, enabling low-level expression of functional TCRαβ. This residual expression likely explains the milder phenotype and extended survival observed in patients carrying this variant. In conclusion, we clarify the nonredundant role of αβ T cells in humans. Our findings show that complete TCRα deficiency causes a SCID-like clinical presentation, whereas partial TCRα deficiency is associated with milder clinical outcomes and longer survival.

## Teaser

Previous reports suggested that inherited TCRα deficiency is not as severe as expected. We report children with complete TCRα deficiency, a lack of αβ T cells, and a severe condition. Previously characterized TCRα deficiency was partial, as opposed to complete.

## Introduction

Human T cell receptors (TCRs) consist of an α and β chain or a γ and δ chain (1). Autosomal recessive (AR) TCRα chain deficiency, resulting from rare variants of the TCRα constant chain (*TRAC*), has been reported in six patients from four unrelated families from Pakistan (2) and India (Table 1) (3, 4). Morgan et al. reported two patients with recurrent viral and bacterial infections who underwent hematopoietic stem cell transplantation (HSCT) at the ages of 6 and 7 years (2). Rawat et al. described three siblings with recurrent respiratory infections who died at the ages of 11 mo and 8 and 11 years (3). These five patients were all homozygous for the same variant, c.*+1G>A, a substitution of the last nucleotide of exon 3, downstream from the translation stop codon. Reverse transcriptase polymerase chain reaction (RT-PCR) on lymphocyte mRNA showed that this mutation caused the skipping of exon 3. This abnormal splicing removes part of the connecting domain and the entire transmembrane and cytoplasmic domains of TCRα (p.Trp107Leufs56*). Despite the lack of conventional CD3^+^TCRαβ^+^ cells in the blood, these patients had γδ^−^CD3^lo^CD4^+^ cells expressing “extremely low levels” of TCRαβ. The authors nevertheless concluded that despite the immune dysregulation and autoimmunity caused by the absence of TCRαβ^+^ T cells, the affected individuals had a surprisingly high level of protection against infection. This finding was surprising because it suggested that αβ T cells were partially redundant, with TCRα deficiency underlying a condition clinically more similar to combined immunodeficiency (CID) than to severe CID (SCID) (5).

**Table 1.** Clinical presentation of TRAC-deficient patients

	This report		Garkaby et al. (4)	Morgan et al. (2)		Rawat et al. (3)		
	Kindred A	Kindred B	Kindred C	Kindred D	Kindred E	Kindred F		
	P1	P2	P3	P4	P5	P6	P7	P8
Mutation cDNA protein	c.192_205del, p.Trp65*	c.361C>T, p.Arg121*	c.347C>G, p.Ser116*	c.*+1G>A, p.Trp107Leufs56*				
TCRα deficiency	Complete	Complete	Complete	Partial	Partial	Partial	Partial	Partial
Clinical diagnosis	SCID	SCID	SCID?	CID	CID	CID	CID	CID
Age at onset	20 days old	<1 mo old	-	15 mo old	6 mo old	3 mo old	6 mo old	<11 mo old
Clinical symptoms	Fever + myocarditis, BCG-osis, CMV infection	Chronic diarrhea, oral thrush, BCG-osis, pneumonia (*Salmonella*), failure to thrive	None, detection by newborn screening	Recurrent respiratory tract infection, otitis media, candidiasis, diarrhea, and failure to thrive. Chronic course of varicella at age 6 as well as chronic EBV and human herpesvirus 6 viremia. Vitiligo and alopecia areata	Recurrent respiratory tract infection, otitis media, candidiasis, diarrhea, and failure to thrive. Eczema and autoimmune hemolytic anemia. Pityriasis rubra pilaris	Recurrent respiratory tract infections, bronchiectasis, warts	Recurrent respiratory tract infections, bronchiectasis renal abscess, warts	Recurrent pneumonia, B cell lymphoma (EBV+)
Age at death or HSCT	HSCT at 22 mo	Death at 3 years old (pneumonia)	HSCT <1 year old	HSCT at 6 years old	HSCT at 7 years old	Death at 11 years old (severe pneumonia)	Death at 8 years old (pulmonary complications)	Death at 11 mo old
Outcome of HSCT	Successful	-	Successful	Successful	Successful	-	-	-
Other relevant information	None	Two siblings died of infection at 5 and 9 mo old, respectively	Sister died at 8 mo old with recurrent bacterial infections, oral thrush, diarrhea, and failure to thrive	None	None	None	None	None

In a third report, Garkaby et al. described an asymptomatic individual identified through SCID newborn screening with low T cell receptor excision circle (TREC) levels. The newborn, a boy, was homozygous for a nonsense mutation (c.347C>G; p.Ser116*) predicted to remove part of the transmembrane and intracellular domains, although the variant was not tested in vitro (4). The patient had low CD3^+^ T cell counts, but αβ and γδ T-cell immunophenotyping was not performed. He underwent HSCT before the occurrence of infection. His older sister had died at 8 mo of age after recurrent infections and poor growth suggestive of a SCID-like condition.

## Results

**Figure 1.**
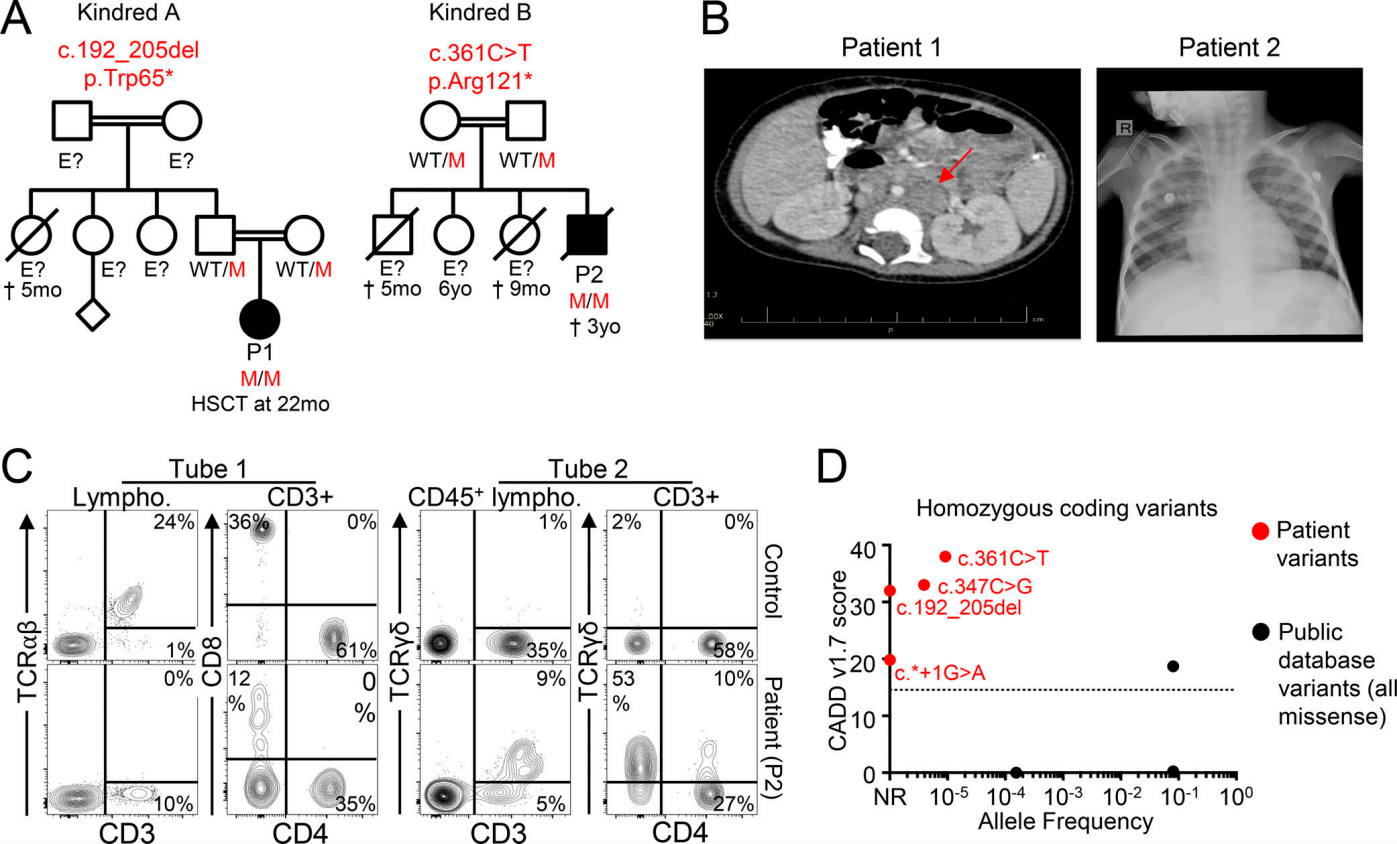
**AR TRAC deficiency. (A)** Pedigree of two Iranian families showing the familial segregation of mutant *TRAC* alleles, labeled as “M.” E?: unknown genotypes. mo, months old; yo, years old. **(B)** Left, spiral abdominopelvic CT scan demonstrating the presence of a large lymph node in the left paravertebral area (18 × 12 mm) and multiple small nonsignificant adenopathies consistent with intranodal abscesses following BCG-osis. Right, chest X-ray imaging revealing bilateral pneumonia in P2 (3 years old). **(C)** Flow cytometry analysis of peripheral blood lymphocytes from P2 (3 mo old) and an aged-matched control. **(D)** Frequency and combined annotation–dependent depletion (CADD) scores for variants present in the homozygous state in the gnomAD database (black dots) and for the *TRAC* mutations reported here or in previous studies (red dots).

We describe two patients from unrelated consanguineous Iranian families with a SCID-like clinical presentation (Fig. 1, A and B; and Table 1, case reports in Materials and methods). P1, a girl, presented recurrent cytomegalovirus (CMV) disease and disseminated *Bacillus* Calmette–Guérin (BCG) infection (BCG-osis) after vaccination, with an onset of disease at the age of 20 days. At 22 mo, she underwent successful HSCT. P2 was a boy with chronic diarrhea and persistent oral thrush from early infancy. At the age of 7 mo, he was diagnosed with BCG-osis. He died from *Salmonella* infection at the age of 3 years. Two of his siblings had died from infections at the ages of 5 and 9 mo. Immunophenotyping for these two patients revealed normal natural killer (NK) and B cell levels, a predominance of TCR-γδ^+^ T cells, and a complete absence of TCR-αβ^+^ T cells (Fig. 1 C, case reports in Materials and methods). Whole-exome sequencing (WES) and Sanger sequencing revealed homozygosity for variants of *TRAC* predicted to be loss-of-function (pLOF) in both patients. P1 is homozygous for a small frameshift deletion (c.195_208del), leading to a premature stop codon (p.Trp65*) (Fig. 1, A and D). P2 was homozygous for another substitution (c.361C>T), leading to a premature stop codon (p.Arg121*) (Fig. 1 A). The c.195_208del variant is private to the family, whereas the c.361C>T variant has an allele frequency of 0.000009136 in gnomAD v4.1 (Fig. 1 D).

**Figure 2.**
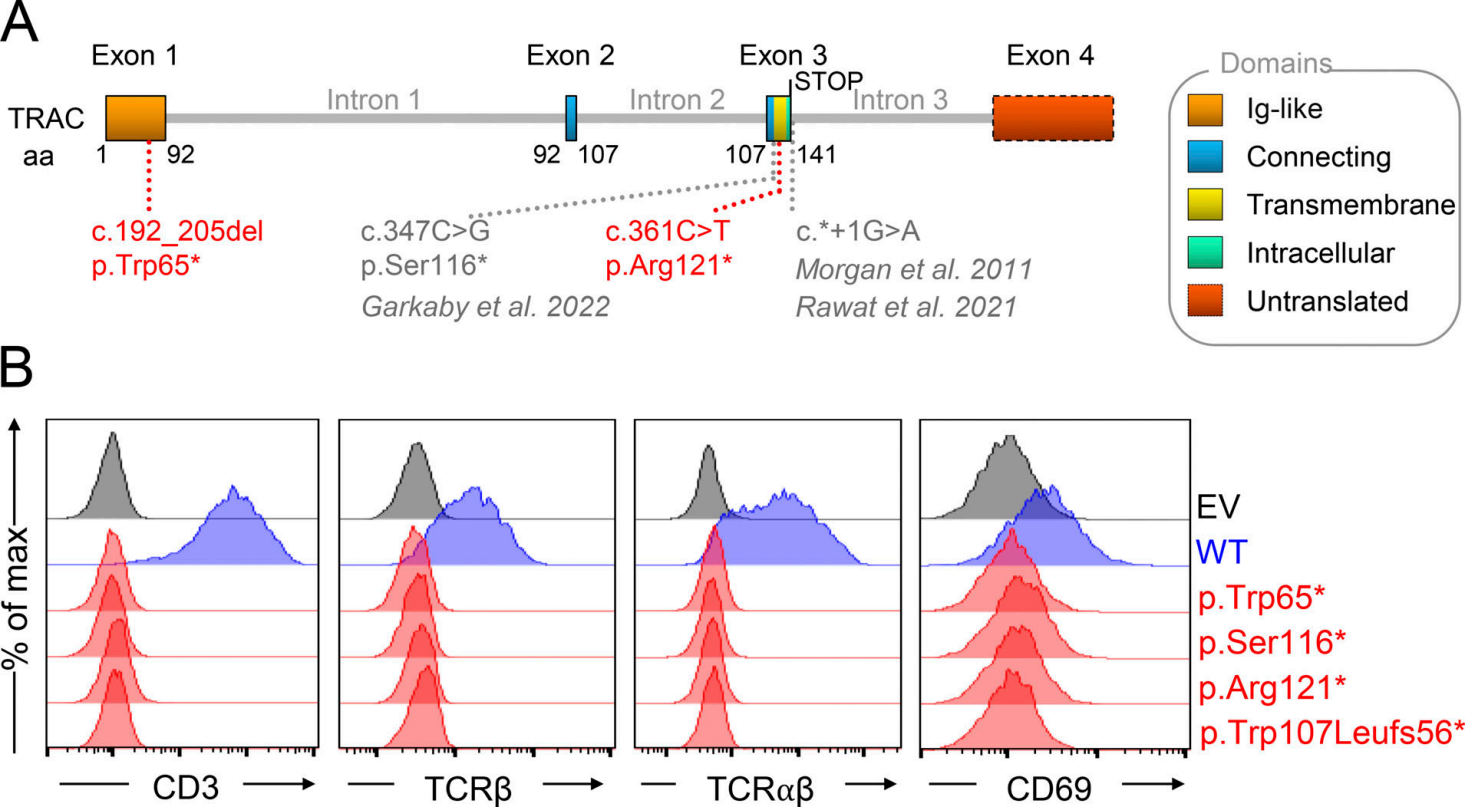
**Patients’ variants are all LOF in the canonical coding sequence of TRAC. (A)** Schematic representations of the *TRAC* gene, with the newly identified mutations in red and previously published mutations in gray. The protein domains are indicated. **(B)** Cell surface expression of CD3ε, TCRβ, TCRαβ, or CD69 evaluated by flow cytometry on TCRα-deficient Jurkat cells transduced with an empty plasmid (EV) or with a plasmid encoding the WT or the indicated TRAC variant in TCRα.

The three *TRAC* alleles underlying TCRα deficiency in patients with a SCID-like clinical phenotype result in premature stop codons in the Ig-like or transmembrane domains (Fig. 2 A). We used in vitro assays to assess the impact of these pLOF variants on protein function. We used TCRα-deficient JR3.11 Jurkat cells transduced with an empty vector (EV) or vectors containing the TCRα cDNA with the wild-type (WT) or mutant *TRAC* variants. We used flow cytometry to evaluate the ability of the constructs to stabilize CD3ε, TCRβ, and TCRαβ on the cell surface and to induce a tonic signal (CD69 induction). Transduction with the WT *TRAC* restored the expression of a functional TCRαβ complex on the cell surface, whereas the p.Trp65*, p.Ser116*, and p.Arg121* variants failed to restore the surface expression of CD3ε, TCRβ, TCRαβ, or the CD69 activation marker (Fig. 2 B). These three *TRAC* variants were therefore LOF in this assay.

**Figure 3.**
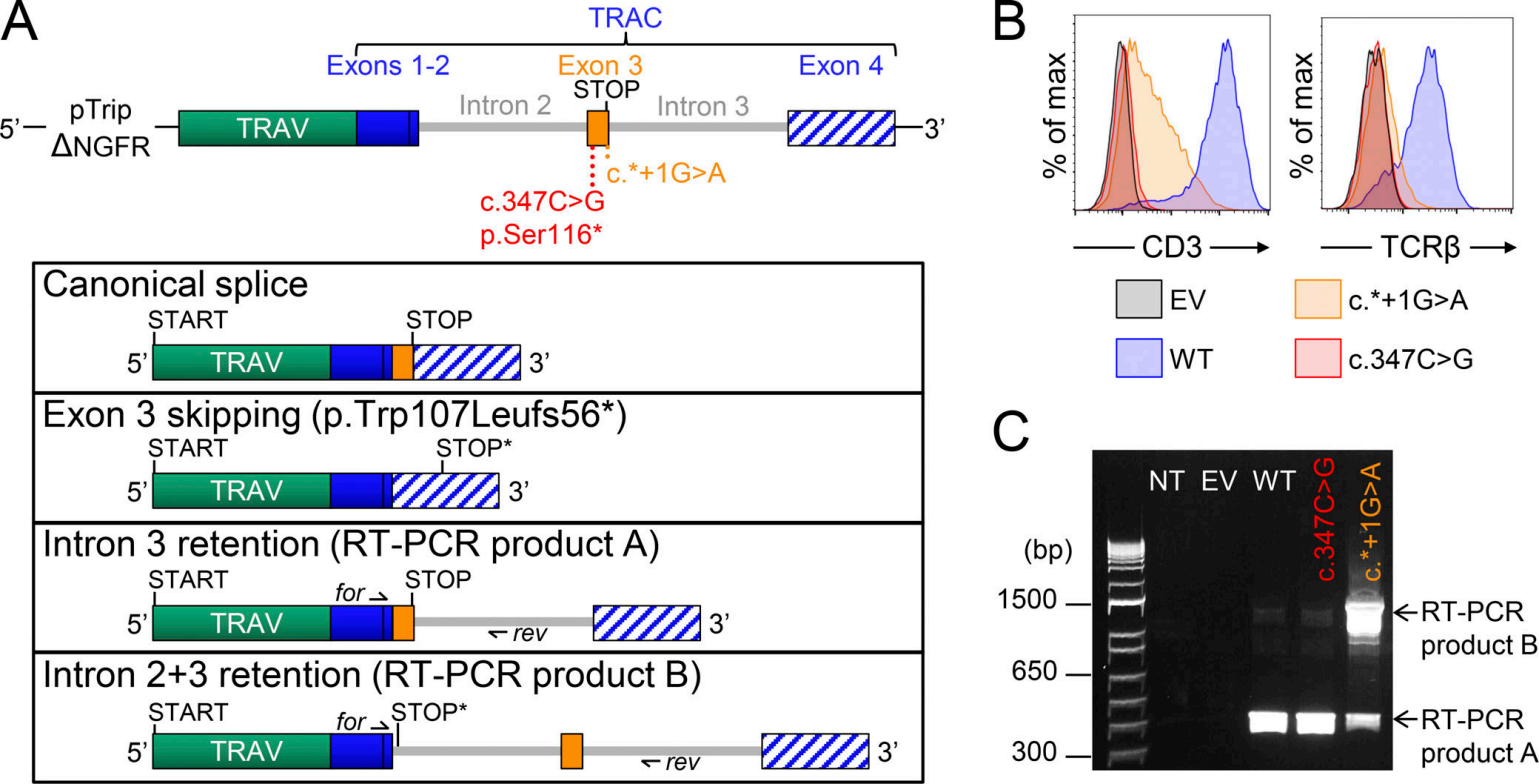
***TRAC* splicing and transcripts activity. (A)** Schematic representation of the artificial TCRα gene created to study the splicing between exons 2, 3, and 4 of *TRAC*. The two mutations tested are depicted together with the various splice variants. STOP*: noncanonical STOP codons generated by alternative splicing. **(B and C)** TCRα-deficient Jurkat cells transduced with EV or a plasmid containing an artificial TCRα gene described in A encoding the WT or the indicated variant of TRAC in TCRα. **(B)** Cell surface expression of CD3ε and TCRβ evaluated by flow cytometry. **(C)** RT-PCR analysis of *TRAC* with primers binding to exon 2 (forward; for) and intron 3 (reverse; rev). NT: Non-transduced. The data shown are representative of three independent experiments. Source data are available for this figure: SourceData F3.

The fourth *TRAC* allele, c.*+1G>A, underlies TCRαβ deficiency in patients with a milder CID-like clinical phenotype (2). It causes abnormal splicing, producing the p.Trp107Leufs56* variant of TRAC. Patients with this mutation survive longer without HSCT and have a T cell subset expressing CD4 and low levels of CD3 and TCRαβ (2, 3). We therefore hypothesized that the c.+1G>A variant is hypomorphic rather than LOF and that the presence of low levels of TCRαβ may confer some protective immunity. We tested this hypothesis by transducing TCRα-deficient JR3.11 Jurkat cells with a cDNA corresponding to the p.Trp107Leufs56* allele (resulting from exon 3 skipping), which failed to restore a functional TCR complex on the cell surface (Fig. 2 B). We then generated an artificial gene containing the variable region of TCRα (TRAV), all 4 *TRAC* exons, and introns 2 and 3 on either side of exon 3 of *TRAC* (Fig. 3 A). This enabled us to test the impact of the noncoding c.+1G>A allele relative to the WT and p.Ser116* alleles in TCRα-deficient JR3.11 Jurkat cells. As in the previous assay, comparison with the WT allele showed that the p.Ser116* construct failed to stabilize a TCR complex on the cell surface (Fig. 3 B). Cells transduced with the c.+1G>A construct displayed a partial stabilization of CD3ε and TCRβ on the surface (Fig. 3 B). PCR on cDNA from transduced cells suggested that the residual activity of the c.+1G>A allele was due to the retention of intron 3 only, preserving the full canonical WT-coding sequence (Fig. 3, A and C). Thus, given the presence of a CD3^lo^TCRαβ^lo^ subset and a CID-like clinical phenotype in the patients (2, 3), our data indicate that the c.+1G>A variant is hypomorphic.

## Discussion

We describe here two patients with complete TCRα deficiency and a SCID-like clinical presentation. We also show that the previously reported c.+1G>A variant is hypomorphic and associated with partial TCRα deficiency, accounting for homozygotes having a CID-like condition (2, 3). An isolated lack of αβ T cells appears to underlie a condition as severe as SCID that is not compensated by other leukocyte subsets, including γδ T cells. By inference, the deficiency of human αβ T cells is probably responsible for most of the severe infections seen in children with SCID and a lack of all types of autologous T cells. Although patients with TRAC deficiency should be detected by SCID newborn screening due to low TREC levels (4), they may not always exhibit the typical immunological features of SCID as defined by the Primary Immune Deficiency Treatment Consortium (6). P1’s total T cell count was >1,000 cells/μl due to a major γδ T cell expansion, far exceeding the cutoff values for SCID diagnosis. To prevent delays in diagnosing TRAC deficiency, TCRαβ and TCRγδ should be assessed by flow cytometry in suspected SCID cases.

## Materials and methods

### Case report of P1

**Figure S1.**
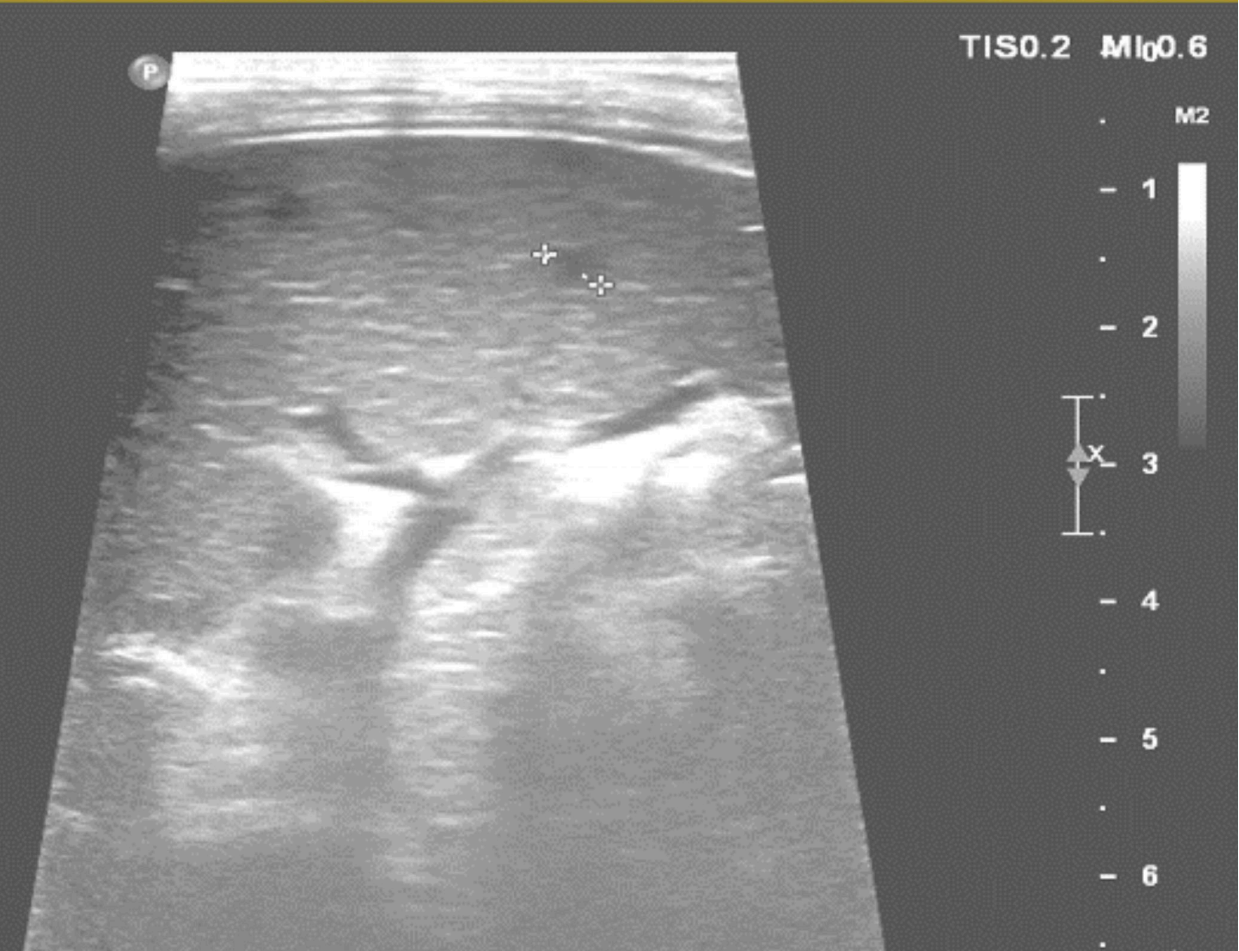
Abdominal ultrasound scan for P1 (4 mo old) showing an enlarged spleen (79-mm diameter) with multiple ill-defined hypoechoic areas (with a maximum size of 4 mm) in the parenchyma.

A 4-mo-old girl was referred to the Children’s Medical Center of Teheran following prolonged fever and gastroenteritis after vaccination with BCG. She was born to consanguineous parents in 2018, at 38 wk, by cesarean section, with no major prenatal or delivery complications. Her parents and both set of grandparents are first cousins with no known family history of an immunodeficiency disorder. P1 was vaccinated with BCG at birth. She had a history of prolonged fever of unknown origin (at the age of 20 days) and myocarditis before hospital admission. At about 1.5 mo of age, she developed a nodule in her left armpit that gradually enlarged to form an abscess with fistulation and pus discharge. An ultrasound of the armpit revealed heterogeneous hypoechoic lesions (mean size of 28 × 26 mm) consistent with an axillary abscess and mobile pus. Macroscopic assessment of the excised mass revealed adhesive lymphadenopathy with central necrosis. Pathology analyses revealed chronic granulomatous inflammation with positive Ziehl–Neelsen stain suggestive of an infection caused by acid-fast organisms. Abdominal ultrasound revealed an enlarged spleen (79-mm diameter) with multiple ill-defined hypoechoic areas (with a maximum size of 4 mm) in the parenchyma (Fig. S1). Ziehl–Neelsen staining of a bone marrow aspiration smear was negative and revealed an increase in granulopoiesis and a shift to the left with lower levels of erythropoiesis suggestive of a reactive bone marrow responding to inflammation. An abdominopelvic CT scan revealed a large lymph node in the left paravertebral area (18 × 12 mm in size) with numerous small adenopathies potentially displaying intranodal abscesses following disseminated BCG infection (BCG-osis) and small low-attenuation splenic foci (Fig. 1 B, left picture). The patient was diagnosed with BCG-osis. Treatment with a four-drug antimycobacterial regimen (isoniazid, rifampicin, ethambutol, and streptomycin), vitamin B6, and interferon-γ was initiated. At the age of 5 mo, P1 presented recurrent fever without identification of the causal pathogen. The patient also experienced several episodes of CMV infection, which was treated with ganciclovir and then acyclovir.

Immunological screening tests and WES were performed, under the assumption that P1 had an inborn error of immunity (Table S1). The patient lacked αβ T cells but had normal γδ T cells, B cells, and NK cells. Based on the identification of a predicted LOF TRAC variant by WES (c.192_205del; p.Trp65*) and the absence of TCRαβ^+^ T cells on flow cytometry analysis, the patient was treated by HSCT. At the age of 22 mo, she received unmanipulated peripheral blood stem cells mobilized by G-CSF from the peripheral blood of a fully matched 15-year-old male cousin (mononucleated cells 7 × 10^8^/kg, CD34^+^ cells 15 × 10^6^/kg, and CD3^+^ cells 235 × 10^6^/kg). The myeloablative conditioning regimen included fludarabine, anti-thymocyte globulin, and melphalan. Treatment against BCG-osis (rifampin, isoniazid, and vitamin B6) was maintained during transplant. The transplantation was successful, but P1 was hospitalized due to various post-HSCT complications. Fluorescence in situ hybridization studies 2 wk and 1 mo after HSCT revealed a 100% donor chimerism (all blood cells were XY). Engraftment of neutrophils (>1,000 cells/mm^3^) occurred on day 10 after HSCT. However, as of her last follow-up, her platelet levels had not yet returned to normal (<50 × 10^3^/μl). Three days after her discharge from the bone marrow transplantation ward, she was admitted because of epistaxis and vomiting (platelets: 12,000; hemoglobin: 8.8). At this time, she was taking isoniazid, rifampin, voriconazole, valganciclovir, cyclosporine, nystatin, and vitamin B6. Three days later, she became pyretic. Her platelet level had decreased to 2,000 and hemoglobin to 7. Blood culture revealed *Pseudomonas aeruginosa* infection, and bronchoalveolar culture was positive for *Klebsiella pneumoniae*. CMV viral load was 14,000 copies/ml. She received antibiotics and anti-viral treatments and was discharged after negative blood cultures. Her subsequent CMV viral loads were 8,923; 4,124; and 335 copies/ml. She had to receive platelet transfusion for 6 mo after her HSCT. 9 mo after HSCT, she was affected with cutaneous graft-versus-host disease and treated until her last follow-up by photopheresis. She had multiple episodes of hospital admission because of fever, gastroenteritis, and low platelet levels. At 5 years old, she was diagnosed with osteoporosis, possibly secondary to a chronic use of corticosteroids. At her last follow-up, she was receiving pamidronate (for osteoporosis), intravenous immunoglobulins (IVIG), ruxolitinib, tacrolimus, ursodeoxycholic acid, and folic acid. The patient is now 6 years old.

### Case report of P2

P2 was an Iranian boy born to distantly related parents. He received BCG and oral polio vaccines at birth. From early infancy, he suffered from chronic diarrhea and persistent oral thrush. At the age of 7 mo, he was diagnosed with BCG-osis and treatment with a regimen of antimycobacterial therapy, trimethoprim-sulfamethoxazole prophylaxis, and monthly IVIG infusions was initiated. At 3 years of age, he presented with protracted diarrhea and severe pneumonia (Fig. 1 B, right picture), leading to admission to the pediatric intensive care unit (PICU) due to failure to thrive, severe dehydration, and respiratory and cardiac failure. Blood and stool cultures did not identify specific pathogens except *Salmonella* spp. Immunological investigations were performed (Table S2). P2 lacked αβ T cells but had normal γδ T cells, B cells, and NK cells. He died 15 days after admission to the PICU due to progressive respiratory failure that did not respond to medical management. Two of his siblings had died from infection with uncharacterized pathogens in early infancy: a brother with mouth ulcers who died from sepsis at 5 mo of age and a sister who died from lung infections at the age of 9 mo. WES data revealed homozygosity for predicted LOF variants of *TRAC* (c.361C>T; p.Arg121*).

### WES

DNA was extracted and sent to Macrogen for WES. Exome analysis was performed as previously described (7).

### Flow cytometry on cells from P2

Fluorescence-activated cell sorting (FACS) analysis was performed on freshly harvested blood cells for routine clinical investigation. Lymphocyte subsets were analyzed in tubes with three to four antibodies conjugated to different fluorochromes. Tube 1: anti–TCRαβ-FITC, anti–CD8-PE, anti–CD3-PerCP-Cy5.5, and anti–CD4-APC. Tube 2: anti–TCRγδ-FITC, anti–CD3-PE, anti–CD45-PerCP-Cy5.5, and anti–CD4-APC. Tube 3: anti–CD16-FITC, anti–CD56-PE, and anti–CD45-PerCP-Cy5.5. Tube 4: anti–CD20-FITC, anti–CD19-PE, and anti–CD45-PerCP-Cy5.5. Erythrocytes were lysed in FACS lysis solution (Becton Dickinson). At least 20,000 leukocytes were analyzed on a BD FACSCanto cytometer (Becton Dickinson). Data were analyzed with FlowJo 10.5.3 software.

### Cell culture

HEK293T cells were maintained in Dulbecco’s modified Eagle medium (no. 61965059; Gibco) supplemented with 10% fetal bovine serum (Sigma-Aldrich). TCRα-deficient JR3.11 Jurkat cells were cultured in RPMI medium (no. 61870044; Gibco) also supplemented with 10% fetal bovine serum. All cell cultures were incubated at 37°C under an atmosphere containing 5% CO_2_. For transfection, HEK293T cells were used to seed 6-well plates at a density of 8 × 10^5^ cells per well.

### Plasmids and transduction

Lentiviral plasmids (pTrip-SFFV-ΔNGFR) encoding TCRα (WT or variants of interest) or the artificial TCRα gene containing introns 2 and 3 and exon 4 of *TRAC* were synthesized by TwistBioscience after onboarding of our EV plasmid. HEK293T cells were transiently transfected with pCMV-VSV-G (0.2 μg), pHXB2 env (0.2 μg; NIH-AIDS Reagent Program; no. 1069), psPAX2 (1 μg; Addgene; no. 12260, gift from D. Trono), and the target vector for transduction, with the X-tremeGENE 9 DNA Transfection Reagent (no. 6365787001; Roche) in accordance with the manufacturer’s guidelines. The medium was replaced after 6 h of incubation. Viral supernatants were collected 24 h later and passed through a filter with 0.2-μm pores. Protamine sulfate (8 μg/ml) was added to the viral supernatant, which was then used to transduce TCRα-deficient JR3.11 Jurkat cells immediately after seeding. Cells were spinoculated at 1,200 × *g* and 25°C for 2 h, then cultured for 48 h at 37°C under an atmosphere containing 5% CO_2_, without shaking. Transduction efficiency was assessed by flow cytometry with an anti-CD271 antibody (no. 557196; 1:500; BD). Enrichment in transduced cells was achieved with a MACS Column and the CD271 MicroBead Kit (no. 130-099-023; Miltenyi Biotec), according to the manufacturer’s protocol.

### Sequence of the WT TCRα cDNA

5′-ATG​TGG​GGA​GTT​TTC​CTT​CTT​TAT​GTT​TCC​ATG​AAG​ATG​GGA​GGC​ACT​ACA​GGA​CAA​AAC​ATT​GAC​CAG​CCC​ACT​GAG​ATG​ACA​GCT​ACG​GAA​GGT​GCC​ATT​GTC​CAG​ATC​AAC​TGC​ACG​TAC​CAG​ACA​TCT​GGG​TTC​AAC​GGG​CTG​TTC​TGG​TAC​CAG​CAA​CAT​GCT​GGC​GAA​GCA​CCC​ACA​TTT​CTG​TCT​TAC​AAT​GTT​CTG​GAT​GGT​TTG​GAG​GAG​AAA​GGT​CGT​TTT​TCT​TCA​TTC​CTT​AGT​CGG​TCT​AAA​GGG​TAC​AGT​TAC​CTC​CTT​TTG​AAG​GAG​CTC​CAG​ATG​AAA​GAC​TCT​GCC​TCT​TAC​CTC​TGT​GCA​GGG​GTG​GAT​AGC​AGC​TAT​AAA​TTG​ATC​TTC​GGA​GCA​GGA​ACA​AGA​CTA​TTT​GTT​AAA​GCA​AAT​ATC​CAG​AAC​CCT​GAC​CCT​GCC​GTG​TAC​CAG​CTG​AGA​GAC​TCT​AAA​TCC​AGT​GAC​AAG​TCT​GTC​TGC​CTA​TTC​ACC​GAT​TTT​GAT​TCT​CAA​ACA​AAT​GTG​TCA​CAA​AGT​AAG​GAT​TCT​GAT​GTG​TAT​ATC​ACA​GAC​AAA​ACT​GTG​CTA​GAC​ATG​AGG​TCT​ATG​GAC​TTC​AAG​AGC​AAC​AGT​GCT​GTG​GCC​TGG​AGC​AAC​AAA​TCT​GAC​TTT​GCA​TGT​GCA​AAC​GCC​TTC​AAC​AAC​AGC​ATT​ATT​CCA​GAA​GAC​ACC​TTC​TTC​CCC​AGC​CCA​GAA​AGT​TCC​TGT​GAT​GTC​AAG​CTG​GTC​GAG​AAA​AGC​TTT​GAA​ACA​GAT​ACG​AAC​CTA​AAC​TTT​CAA​AAC​CTG​TCA​GTG​ATT​GGG​TTC​CGA​ATC​CTC​CTC​CTG​AAA​GTG​GCC​GGG​TTT​AAT​CTG​CTC​ATG​ACG​CTG​CGG​CTG​TGG​TCC​AGC​TGA-3′.

### Sequence of the artificial TCRα WT cDNA with introns 2 and 3 and exon 4 of *TRAC*

5′-ATG​TGG​GGA​GTT​TTC​CTT​CTT​TAT​GTT​TCC​ATG​AAG​ATG​GGA​GGC​ACT​ACA​GGA​CAA​AAC​ATT​GAC​CAG​CCC​ACT​GAG​ATG​ACA​GCT​ACG​GAA​GGT​GCC​ATT​GTC​CAG​ATC​AAC​TGC​ACG​TAC​CAG​ACA​TCT​GGG​TTC​AAC​GGG​CTG​TTC​TGG​TAC​CAG​CAA​CAT​GCT​GGC​GAA​GCA​CCC​ACA​TTT​CTG​TCT​TAC​AAT​GTT​CTG​GAT​GGT​TTG​GAG​GAG​AAA​GGT​CGT​TTT​TCT​TCA​TTC​CTT​AGT​CGG​TCT​AAA​GGG​TAC​AGT​TAC​CTC​CTT​TTG​AAG​GAG​CTC​CAG​ATG​AAA​GAC​TCT​GCC​TCT​TAC​CTC​TGT​GCA​GGG​GTG​GAT​AGC​AGC​TAT​AAA​TTG​ATC​TTC​GGA​GCA​GGA​ACA​AGA​CTA​TTT​GTT​AAA​GCA​AAT​ATC​CAG​AAC​CCT​GAC​CCT​GCC​GTG​TAC​CAG​CTG​AGA​GAC​TCT​AAA​TCC​AGT​GAC​AAG​TCT​GTC​TGC​CTA​TTC​ACC​GAT​TTT​GAT​TCT​CAA​ACA​AAT​GTG​TCA​CAA​AGT​AAG​GAT​TCT​GAT​GTG​TAT​ATC​ACA​GAC​AAA​ACT​GTG​CTA​GAC​ATG​AGG​TCT​ATG​GAC​TTC​AAG​AGC​AAC​AGT​GCT​GTG​GCC​TGG​AGC​AAC​AAA​TCT​GAC​TTT​GCA​TGT​GCA​AAC​GCC​TTC​AAC​AAC​AGC​ATT​ATT​CCA​GAA​GAC​ACC​TTC​TTC​CCC​AGC​CCA​GAA​AGT​TCC​TGT​GAT​GTC​AAG​CTG​GTC​GAG​AAA​AGC​TTT​GAA​ACA​GGT​AAG​ACA​GGG​GTC​TAG​CCT​GGG​TTT​GCA​CAG​GAT​TGC​GGA​AGT​GAT​GAA​CCC​GCA​ATA​ACC​CTG​CCT​GGA​TGA​GGG​AGT​GGG​AAG​AAA​TTA​GTA​GAT​GTG​GGA​ATG​AAT​GAT​GAG​GAA​TGG​AAA​CAG​CGG​TTC​AAG​ACC​TGC​CCA​GAG​CTG​GGT​GGG​GTC​TCT​CCT​GAA​TCC​CTC​TCA​CCA​TCT​CTG​ACT​TTC​CAT​TCT​AAG​CAC​TTT​GAG​GAT​GAG​TTT​CTA​GCT​TCA​ATA​GAC​CAA​GGA​CTC​TCT​CCT​AGG​CCT​CTG​TAT​TCC​TTT​CAA​CAG​CTC​CAC​TGT​CAA​GAG​AGC​CAG​AGA​GAG​CTT​CTG​GGT​GGC​CCA​GCT​GTG​AAA​TTT​CTG​AGT​CCC​TTA​GGG​ATA​GCC​CTA​AAC​GAA​CCA​GAT​CAT​CCT​GAG​GAC​AGC​CAA​GAG​GTT​TTG​CCT​TCT​TTC​AAG​ACA​AGC​AAC​AGT​ACT​CAC​ATA​GGC​TGT​GGG​CAA​TGG​TCC​TGT​CTC​TCA​AGA​ATC​CCC​TGC​CAC​TCC​TCA​CAC​CCA​CCC​TGG​GCC​CAT​ATT​CAT​TTC​CAT​TTG​AGT​TGT​TCT​TAT​TGA​GTC​ATC​CTT​CCT​GTG​GTA​GCG​GAA​CTC​ACT​AAG​GGG​CCC​ATC​TGG​ACC​CGA​GGT​ATT​GTG​ATG​ATA​AAT​TCT​GAG​CAC​CTA​CCC​CAT​CCC​CAG​AAG​GGC​TCA​GAA​ATA​AAA​TAA​GAG​CCA​AGT​CTA​GTC​GGT​GTT​TCC​TGT​CTT​GAA​ACA​CAA​TAC​TGT​TGG​CCC​TGG​AAG​AAT​GCA​CAG​AAT​CTG​TTT​GTA​AGG​GGA​TAT​GCA​CAG​AAG​CTG​CAA​GGG​ACA​GGA​GGT​GCA​GGA​GCT​GCA​GGC​CTC​CCC​CAC​CCA​GCC​TGC​TCT​GCC​TTG​GGG​AAA​ACC​GTG​GGT​GTG​TCC​TGC​AGG​CCA​TGC​AGG​CCT​GGG​ACA​TGC​AAG​CCC​ATA​ACC​GCT​GTG​GCC​TCT​TGG​TTT​TAC​AGA​TAC​GAA​CCT​AAA​CTT​TCA​AAA​CCT​GTC​AGT​GAT​TGG​GTT​CCG​AAT​CCT​CCT​CCT​GAA​AGT​GGC​CGG​GTT​TAA​TCT​GCT​CAT​GAC​GCT​GCG​GCT​GTG​GTC​CAG​CTG​AGG​TGA​GGG​GCC​TTG​AAG​CTG​GGA​GTG​GGG​TTT​AGG​GAC​GCG​GGT​CTC​TGG​GTG​CAT​CCT​AAG​CTC​TGA​GAG​CAA​ACC​TCC​CTG​CAG​GGT​CTT​GCT​TTT​AAG​TCC​AAA​GCC​TGA​GCC​CAC​CAA​ACT​CTC​CTA​CTT​CTT​CCT​GTT​ACA​AAT​TCC​TCT​TGT​GCA​ATA​ATA​ATG​GCC​TGA​AAC​GCT​GTA​AAA​TAT​CCT​CAT​TTC​AGC​CGC​CTC​AGT​TGC​ACT​TCT​CCC​CTA​TGA​GGT​AGG​AAG​AAC​AGT​TGT​TTA​GAA​ACG​AAG​AAA​CTG​AGG​CCC​CAC​AGC​TAA​TGA​GTG​GAG​GAA​GAG​AGA​CAC​TTG​TGT​ACA​CCA​CAT​GCC​TTG​TGT​TGT​ACT​TCT​CTC​ACC​GTG​TAA​CCT​CCT​CAT​GTC​CTC​TCT​CCC​CAG​TAC​GGC​TCT​CTT​AGC​TCA​GTA​GAA​AGA​AGA​CAT​TAC​ACT​CAT​ATT​ACA​CCC​CAA​TCC​TGG​CTA​GAG​TCT​CCG​CAC​CCT​CCT​CCC​CCA​GGG​TCC​CCA​GTC​GTC​TTG​CTG​ACA​ACT​GCA​TCC​TGT​TCC​ATC​ACC​ATC​AAA​AAA​AAA​CTC​CAG​GCT​GGG​TGC​GGG​GGC​TCA​CAC​CTG​TAA​TCC​CAG​CAC​TTT​GGG​AGG​CAG​AGG​CAG​GAG​GAG​CAC​AGG​AGC​TGG​AGA​CCA​GCC​TGG​GCA​ACA​CAG​GGA​GAC​CCC​GCC​TCT​ACA​AAA​AGT​GAA​AAA​ATT​AAC​CAG​GTG​TGG​TGC​TGC​ACA​CCT​GTA​GTC​CCA​GCT​ACT​TAA​GAG​GCT​GAG​ATG​GGA​GGA​TCG​CTT​GAG​CCC​TGG​AAT​GTT​GAG​GCT​ACA​ATG​AGC​TGT​GAT​TGC​GTC​ACT​GCA​CTC​CAG​CCT​GGA​AGA​CAA​AGC​AAG​ATC​CTG​TCT​CAA​ATA​ATA​AAA​AAA​ATA​AGA​ACT​CCA​GGG​TAC​ATT​TGC​TCC​TAG​AAC​TCT​ACC​ACA​TAG​CCC​CAA​ACA​GAG​CCA​TCA​CCA​TCA​CAT​CCC​TAA​CAG​TCC​TGG​GTC​TTC​CTC​AGT​GTC​CAG​CCT​GAC​TTC​TGT​TCT​TCC​TCA​TTC​CAG​ATC​TGC​AAG​ATT​GTA​AGA​CAG​CCT​GTG​CTC​CCT​CGC​TCC​TTC​CTC​TGC​ATT​GCC​CCT​CTT​CTC​CCT​CTC​CAA​ACA​GAG​GGA​ACT​CTC​CTA​CCC​CCA​AGG​AGG​TGA​AAG​CTG​CTA​CCA​CCT​CTG​TGC​CCC​CCC​GGC​AAT​GCC​ACC​AAC​TGG​ATC​CTA​CCC​GAA​TTT​ATG​ATT​AAG​ATT​GCT​GAA​GAG​CTG​CCA​AAC​ACT​GCT​GCC​ACC​CCC​TCT​GTT​CCC​TTA​TTG​CTG​CTT​GTC​ACT​GCC​TGA​CAT​TCA​CGG​CAG​AGG​CAA​GGC​TGC​TGC​AGC​CTC​CCC​TGG​CTG​TGC​ACA​TTC​CCT​CCT​GCT​CCC​CAG​AGA​CTG​CCT​CCG​CCA​TCC​CAC​AGA​TGA​TGG​ATC​TTC​AGT​GGG​TTC​TCT​TGG​GCT​CTA​GGT​CCT​GCA​GAA​TGT​TGT​GAG​GGG​TTT​ATT​TTT​TTT​TAA​TAG​TGT​TCA​TAA​AGA​AAT​ACA​TAG​TAT​TCT​TCT​TCT​CAA​GAC​GTG​GGG​GGA​AAT​TAT​CTC​ATT​ATC​GAG​GCC​CTG​CTA​TGC​TGT​GTA​TCT​GGG​CGT​GTT​GTA​TGT​CCT​GCT​GCC​GAT​GCC​TTC​ATT​AAA​ATG​ATT​TGG​AAG​AGC​A-3′.

### Flow cytometry of transduced cells

Transduced JR3.11 Jurkat cells were labeled with antibodies targeting CD3 (no. 555333, 2:50; BD), C1β TCR (no. 565776, 2:50; BD), or CD69 (no. 310912, 1:400; Biolegend). Cells were also treated with the Aqua Live/Dead Cell Stain Kit (Thermo Fisher Scientific) for 1 h at room temperature. Flow cytometry analysis was performed on a Gallios flow cytometer (Beckman Coulter), and all data were analyzed with FlowJo 10.5.3 software.

### mRNA purification and RT-PCR

Total RNA was isolated with the RNeasy Extraction Kit (Qiagen) from TCRα-deficient JR3.11 Jurkat cells transduced with the artificial TCRα genes. The RNA was then reverse transcribed to generate cDNA with the SuperScript II reverse transcriptase (Thermo Fisher Scientific) and oligo-dT primers (Thermo Fisher Scientific). TCRα transcripts were amplified with cloneAmp Hifi premix and polymerase (no. 639298; Takara), with the forward primer 5′-GTG​ATG​TCA​AGC​TGG​TCG​AG-3′ and the reverse primer 5′-ATA​GGG​GAG​AAG​TGC​AAC​TGA​GGC-3′. PCR products were run on a 1% agarose gel for 1 h at 120 V.

### Statistics

No formal statistical analysis was conducted for this study, as such analysis was not compatible with the study design.

### Study approval

Informed consent for participation in this study was obtained in accordance with local regulations, with approval from the institutional review board (IRB). The experiments described here were performed in France, in accordance with local regulations and with the approval of the IRB of Necker Hospital for Sick Children, France. Written informed consent to participate was obtained from the parents of the patient. Consent to publish this report was obtained from the parents of the patients. All the authors approved the final version of the manuscript.

### Online supplemental material

Fig. S1 shows an abdominal ultrasound image of P1. Tables S1 and S2 present the laboratory data of P1 and P2, respectively.

## Supplementary Material

Table S1 shows the laboratory data for patient 1 before HSCT.

Table S2 shows the laboratory data for patient 2.

SourceData F3 is the source file for Fig. 3.

## Data Availability

Data supporting the findings of this study, including values underlying the graphical data and the means reported in the main text and supplemental material, are available from the corresponding author upon reasonable request. Deidentified human subject data can be provided where permitted. No large datasets or computational code requiring deposition in public repositories were generated during this study.
