# Supplementary material for: Two different forms of inherited human TCRα chain deficiency
Source: J Hum Immun. 2025 Jun 4;1(2):e20250014. doi: 10.70962/jhi.20250014 (PMC12526356; doi:10.70962/jhi.20250014)
Supplement: Table S1 — shows the laboratory data for patient 1 before HSCT. [file jhi_20250014_tables1.docx]

**Table S1**. Laboratory data for patient 1 before HSCT

|  | **4 months** | **12 months** | **Normal range**  **(6-24 months old)** |
| --- | --- | --- | --- |
| **WBC (***10^3^/μL**)** | 11.36 | 27.84 | 6-17 |
| **RBC (***10^6^/μL**)** | 4.16 | 3.75 | 3.7-5.3 |
| **Hb (**g/dL**)** | 8.4 | 6.2 | 10.5-13.5 |
| **Platelets (***10^3^/μL**)** | 316 | 531 | 150-450 |
| **Neutrophils**  % of WBC  Count (*10^3^/μL) | 64.5  7.33 | 66.7  18.75 | 15-35  1-8.5 |
| **Lymphocytes**  % of WBC  Count (*10^3^/μL) | 25.7  2.92 | 25  6.97 | 45-76  4-12 |
| **Monocytes**  % of WBC  Count (*10^3^/μL) | 9.6  1.09 | 7.3  2.02 | 4-12  0.2-1.1 |
| **Eosinophils**  % of WBC  Count (*10^3^/μL) | 0.1  0.01 | 0.9  0.26 | 0-3  <0.8 |
| **Basophils**  % of WBC  Count (*10^3^/μL) | 0.1  0.01 | 0.02  0.02 | 0-1  <0.2 |
|  |  |  |  |
| **Flow cytometry** | Date: 10/18/2018 | 06/30/2019 |  |
| **Granulocytes (%)** | 69 | 72 |  |
| **Monocytes (%)** | 7 | 5 |  |
| **Lymphocytes (%)** | 24 | 23 |  |
| **CD3^+^** 10^3^/μL (% of lympho.) | 1314 (45) | 5576 (80) | 2200-9200 |
| **CD19^+^** 10^3^/μL (% of lympho.) | 1250 (43) | 627 (9) | 500-2300 |
| **CD20^+^** 10^3^/μL (% of lympho.) | 1250 (43) |  | 500-2300 |
| **CD16^+^** 10^3^/μL (% of lympho.) | 350 (12) |  | 97-1990 |
| **CD56^+^** 10^3^/μL (% of lympho.) | 350 (12) | 766 (11) | 97-1990 |
|  |  |  |  |
| **NBT 98%** | 98% |  | 90-100 |
| **Anti-tetanus 1.2** | 1.2 |  |  |
| **Anti-diphtheria 0.1** | 0.1 |  |  |
| **C3** | 126 |  | 64-167 |
| **C4** | 27 |  | 7.1-36 |
| **CH50** | 134 |  | 70-150 |
| **Coombs Direct** | Negative |  |  |
|  |  |  |  |
| **TCR panel (% of CD3^+^)** |  | 30/6/2019 |  |
| **CD3^+^CD4^+^** |  | 2 |  |
| **CD3^+^CD8^+^** |  | 25 |  |
| **CD3^+^TCR-αβ^+^** |  | 0 | 90-100% |
| **CD3^+^TCR-γδ^+^** |  | 95 | 1-10% |
| **CD4^+^TCR-αβ^+^** |  | 0 |  |
| **CD4^+^TCR-γδ^+^** |  | 36 |  |
| **CD3^+^αβ^+^CD4^-^CD8^-^** |  | 0 | <2% |
|  |  |  |  |
| **CSF analysis** | WBC 3 PMNs, RBC 3680 (70% dysmorphic), no bacteria |  |  |
| **CSF culture** | Negative |  |  |
| **CSF Smear** | No acid-fast bacilli |  |  |
| **Abscess discharge smear** | Positive for acid-fast bacilli,  No bacteria on gram staining,  60% PMNs |  |  |
| **Abscess culture** | Negative |  |  |
| **Bone-marrow culture** | Negative |  |  |
