# Supplementary material for: Two different forms of inherited human TCRα chain deficiency
Source: J Hum Immun. 2025 Jun 4;1(2):e20250014. doi: 10.70962/jhi.20250014 (PMC12526356; doi:10.70962/jhi.20250014)
Supplement: Table S2 — shows the laboratory data for patient 2. [file jhi_20250014_tables2.docx]

**Table S2**. Laboratory data for patient 2

|  | **7 months** | **2 years** | **3 years** | **Normal ranges** |
| --- | --- | --- | --- | --- |
| **WBC (cells/μL)** | 8,320 | 4,600 | 7,320 | 4-10 |
| **Lymphocytes** | 1,080 | 900 | 1,630 | 800-4,000 |
| **Neutrophils** | 6,330 | 2,200 | 4,370 | 2,000-7,000 |
| **Monocytes** | 410 | 1,200 | 1,000 | 120-800 |
| **Hb (g/dL)** | 7.6 | 11 | 10 | 11-16 |
| **RBC (millions)** | 2.64 | 3.2 | 3.39 | 3.5-5.5 |
| **MCV (fL)** | 88 | 92 | 89 | 80-100 |
| **Platelets (cells/μL)** | 313,000 | 170,000 | 499,000 | 150,000-450,000 |
|  |  |  |  |  |
| **CRP mg/L** | - | 15 | 12 | <6 |
| **ESR mm/h** | - | 37 | 36 | 0-10 |
| **IgG (mg/dL)** | 220 | 578 (on IVIG) | - |  |
| **IgM (mg/dL)** | 45 | - | - |  |
| **IgA (mg/dL)** | undetectable | - | - |  |
|  |  |  |  |  |
| **CD45 %** | - | - | 100 |  |
| **CD3 %** | - | - | 15 |  |
| **CD4 %** | - | - | 7 |  |
| **CD8 %** | - | - | 8 |  |
| **CD19 %** | - | - | 74 |  |
| **CD16/56 %** | - | - | 8 |  |
|  |  |  |  |  |
| **CD3^+^** | - | - | 14 | 90-100 |
| **CD3^+^TCRgd^+^** | - | - | 64 | 1-10 |
| **CD3^+^TCRab^+^** | - | - | 0 | 1-99 |
|  |  |  |  |  |
| **CMV viral load** | - | - | Negative |  |
| **EBV viral load** | - | - | Negative |  |
| **SARS-CoV Rt-PCR** | Positive | - | Negative |  |
|  |  |  |  |  |
| **Stool culture** | - | - | *Salmonella* spp. |  |
| **Blood culture** | *-* | Negative | *Salmonella* spp. |  |
