# Supplementary figures and images for: Two different forms of inherited human TCRα chain deficiency
Source: J Hum Immun. 2025 Jun 4;1(2):e20250014. doi: 10.70962/jhi.20250014 (PMC12526356; doi:10.70962/jhi.20250014)

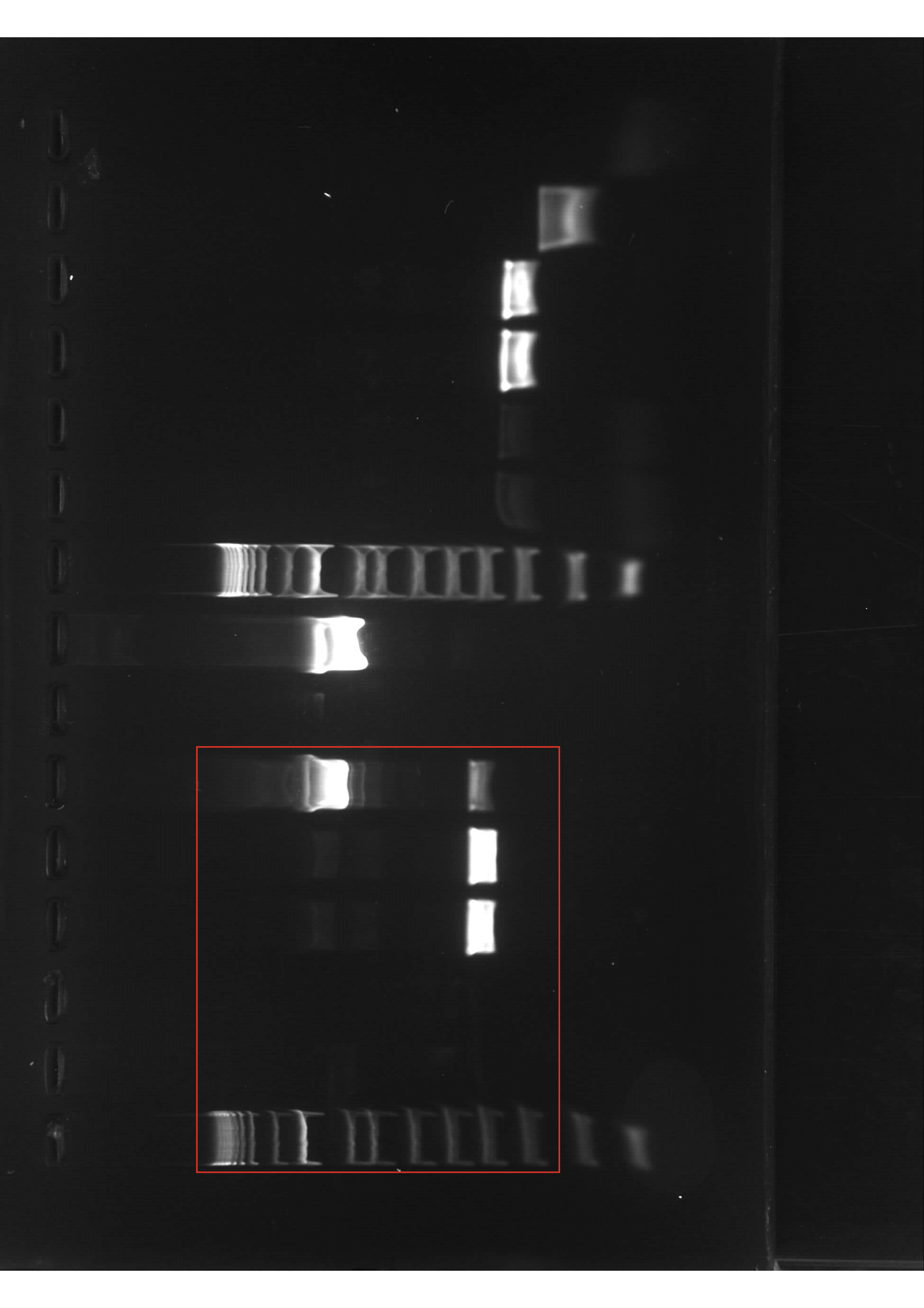

Supplement: SourceData F3 — is the source file for Fig. 3. [file jhi_20250014_sourcedataf3.pdf]
